# Disruptive coloration and binocular disparity: breaking camouflage

**DOI:** 10.1098/rspb.2018.2045

**Published:** 2019-02-13

**Authors:** Wendy J. Adams, Erich W. Graf, Matt Anderson

**Affiliations:** Department of Psychology, University of Southampton, Southampton SO17 1BJ, UK

**Keywords:** camouflage, visual perception, disruptive coloration, binocular disparity, depth, human

## Abstract

Many species employ camouflage to disguise their true shape and avoid detection or recognition. Disruptive coloration is a form of camouflage in which high-contrast patterns obscure internal features or break up an animal's outline. In particular, edge enhancement creates illusory, or ‘fake’ depth edges within the animal's body. Disruptive coloration often co-occurs with background matching, and together, these strategies make it difficult for an observer to visually segment an animal from its background. However, stereoscopic vision could provide a critical advantage in the arms race between perception and camouflage: the depth information provided by binocular disparities reveals the true three-dimensional layout of a scene, and might, therefore, help an observer to overcome the effects of disruptive coloration. Human observers located snake targets embedded in leafy backgrounds. We analysed performance (response time) as a function of edge enhancement, illumination conditions and the availability of binocular depth cues. We confirm that edge enhancement contributes to effective camouflage: observers were slower to find snakes whose patterning contains ‘fake’ depth edges. Importantly, however, this effect disappeared when binocular depth cues were available. Illumination also affected detection: under directional illumination, where both the leaves and snake produced strong cast shadows, snake targets were localized more quickly than in scenes rendered under ambient illumination. In summary, we show that illusory depth edges, created via disruptive coloration, help to conceal targets from human observers. However, cast shadows and binocular depth information improve detection by providing information about the true three-dimensional structure of a scene. Importantly, the strong interaction between disparity and edge enhancement suggests that stereoscopic vision has a critical role in breaking camouflage, enabling the observer to overcome the disruptive effects of edge enhancement.

## 1. Introduction

**Figure 1.**
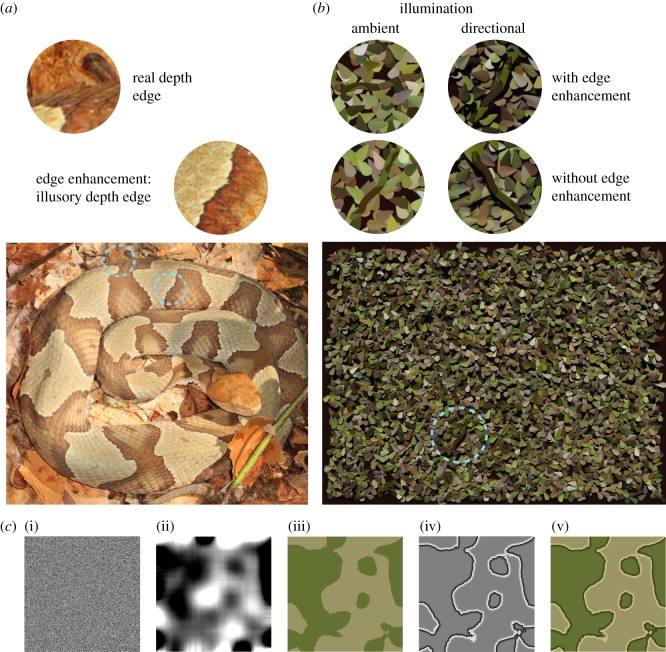
Camouflaged snakes. (*a*) The patterns on the copperhead snake illustrate both edge enhancement and background matching. In natural scenes, humans must discriminate depth edges at object boundaries (left close-up) from edges owing to other causes such as a change in reflectance (right close-up), or a shadow boundary. Image by Judy Gallagher, under licence: https://creativecommons.org/licenses/by/2.0/deed.en. (*b*) Example stimuli. A single snake target was presented on each trial and observers reported its location (one of four quadrants). The four disc close-ups show example targets with or without edge enhancement (rows), in scenes under ambient or directional illumination (columns). (*c*) Generation of snake textures. (i) Random-dot noise patterns were low-pass filtered to create (ii) blobby greyscale patterns. (iii) These patterns were thresholded to create binary maps. Colours from forest scenes within the SYNS dataset [1] were applied to these binary pattern maps, to create snake textures without edge enhancement. (iv) The edge enhancement pattern comprised a linear luminance gradient on each side of the pattern's edges. This enhancement pattern was combined with the base texture (iii) to create (v) the edge-enhanced snake texture. See Methods: Stimuli for more details.

Many animals, such as the copperhead snake (*Agkistrodon contortrix*, figure 1*a*), use camouflage to avoid detection or recognition. Since the classic work of Thayer & Thayer [2] and Cott [3], two distinct camouflage strategies have been characterized: *background matching* and *disruptive coloration*. Both strategies disrupt the visual signals that would normally give away an animal or object's outline shape [4]. Background matching reduces the visibility of an animal's true boundaries: the animal's patterning approximates the luminance, colour and texture of the background, such that it blends into its surroundings [3,5,6]. Although background matching does improve crypsis [7], i.e. the avoidance of detection, in most situations it will provide limited camouflage. Even if regional texture statistics are well matched, local discontinuities can still reveal an animal's outline [2,8]. Moreover, animals may need to occupy multiple, visually distinct habitats. For this reason, it is argued, many species also employ disruptive coloration [2,3]*.*

Disruptive coloration can be defined as patterning that breaks up an animal's surfaces and/or bounding contours, to provide misleading information about its shape (see Stevens & Merilaita [8] for a review). Disruptive coloration often co-occurs with background matching and enhances concealment from various predators, including birds and humans [3,9,10]. Edge enhancement is a form of disruptive coloration in which salient, high-contrast edges within the animal's body separate regions of different luminance and/or hue (figure 1*a*). The enhanced contrast is provided by local luminance gradients adjacent to the reflectance boundary: the light region becomes lighter and the dark region becomes darker (figure 1*c*). This type of disruptive coloration was described by Cott [3], who also suggested that internal edges with enhanced contrast may convey a (misleading) impression of depth. These ‘fake’ depth edges may be particularly effective in thwarting segmentation (i.e. the process of visually separating objects from each other, and from their background) if they are interpreted as part of an animal's bounding contour.

Evidence from frogs supports the idea that edge enhancement evolved as an effective camouflage strategy, i.e. a form of disruptive coloration, rather than as a by-product of bi-coloration, or as a warning signal. First, the Australian *Limnodynastes* frog (one of many frog species to employ edge enhancement) can alter its body colours to match the surroundings [11], but maintains its high-contrast enhanced edges [12]. Second, in contrast to their non-toxic relatives, species such as the poison dart frog (*Dendrobates*) have colourful, high-contrast aposematic patterns that do not feature edge enhancement [12].

Recently, Egan *et al*. [13] confirmed that humans are slower to find targets with edge-enhanced patterns, and perceive depth changes at enhanced edges. A subsequent study revealed that edge enhancement not only affects target detection, but also slows down recognition [14]. Background matching and edge enhancement can be thought of as complementary camouflage strategies: the former disguises the true boundaries of an animal, whereas the latter provides misleading segmentation cues (i.e. fake bounding contours) within the animal's body. Together, these strategies conceal the true bounding contour of a predator or prey to impede segmentation, detection and recognition.

Animals with stereoscopic vision—such as humans—may be harder to hide from. Our inter-ocular separation leads to binocular disparities (small differences between the left and right eyes' images of the scene) that correspond to the scene's true depth structure. Random-dot stereograms (RDSs; Julesz [15]) are classic perceptual stimuli that demonstrate the role of disparity in depth perception. These stimuli contain no pictorial cues, and provide depth information only via binocular disparities. Julesz and others have hypothesized that binocular disparities may have a role in breaking camouflage, noting that, within an RDS, ‘objects jump out in depth when stereoscopically fused’ ([15], pp. 145–46). The veridical depth information provided by stereopsis could make camouflage less effective—enabling the observer to identify true object boundaries and reject ‘false’ edges. Surprisingly, however, little is known about whether binocular disparity enhances the detection of targets—either with or without camouflage—within naturalistic, complex scenes. Camouflage experiments with human observers tend to use monoscopic viewing (both eyes view the same two-dimensional image), and thus lack the binocular depth information that is available in natural viewing.

RDSs differ dramatically from natural scenes: the monocular images lack any coherent structure such as discernible edges or other features. Instead, each eye's image is a collection of pixels with randomly varying luminance (see the random-dot pattern in left-most panel of figure 1*c*). By contrast, natural images include an abundance of features, including salient edges, most of which are not associated with object boundaries. Moreover, camouflage strategies such as edge enhancement create salient image features that imply object boundaries where none truly exist. For the binocular observer to overcome disruptive coloration, binocular disparity must be processed in conjunction with other visual cues to depth. Edge enhancement camouflage pits pictorial depth cues against binocular disparity information.

Further, several authors have noted that long durations are required (seconds or even minutes) for observers to achieve an initial perception of depth from RDS displays [16–18]. In addition, temporal thresholds to perceive disparity-defined depth modulations are an order of magnitude longer than those to perceive luminance modulations [19–23]. This evidence begs the question of whether binocular disparity—an apparently sluggish depth cue—can facilitate efficient visual search.

**Figure 2.**
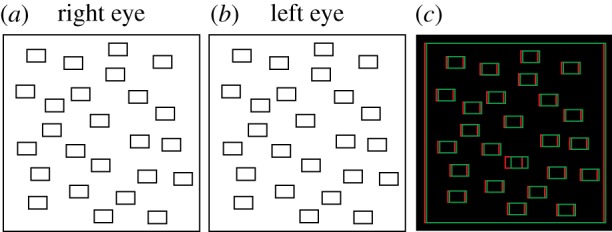
Classic visual search displays used to investigate the effects of binocular disparity. Panels (*a,b*) can be cross-fused to identify the target that is stereoscopically in front of the distractors. (*c*) The same stimulus layout, presented as an anaglyph, for viewing with red-green glasses.

Evidence for the efficacy of binocular disparity in visual search comes from classic search paradigms, employing displays similar to that shown in figure 2. Observers view an array of simple objects and identify the target ‘odd one out’ among distractors. Within such displays, binocular disparity can act as a pre-attentive feature: a target that is stereoscopically in front of a plane of distractors ‘pops out’, i.e. is detected quickly, irrespective of the number of distractors [24–26]. Clearly, such visual stimuli are highly unnatural: individual target and distractor elements are clearly separable; there is no ‘segmentation problem’. In addition, efficient search is dependent on the anomalous three-dimensional layout: the pop-out effect does not extend to other depth configurations, such when the distractors vary in their disparity-defined depth, or the target is presented at a greater depth than the distractors [25].

Caziot & Backus [27] provide one of the few studies that explore the role of disparity when searching within complex, naturalistic images. Observers were asked to identify targets (e.g. birds and planes) within photographs of real street scenes. Performance improved when the target was stereoscopically in front of the background (rather than at the same depth). Thus, the disparity benefit for targets that are presented in front of a planar, or near-planar background appears to extend to more complex environments. This is consistent with work from Finlayson *et al*. [28], showing that search is facilitated when observers can attend to (and thus limit their search to) a known depth plane.

Here we directly probe human observers' ability to find camouflaged targets within a cluttered, three-dimensional environment. We vary the three-dimensional positions of the target and distractors from trial-to-trial, such that the target depth is unknown. We investigate whether edge enhancement impedes search, and whether binocular depth cues facilitate search. Moreover, we ask whether disparity enables observers to overcome disruptive coloration, i.e. to ‘break camouflage’, within naturalistic, complex scenes. In other words, does edge enhancement become less effective when binocular depth cues are available?

We also investigate the effect of illumination conditions. Directional illumination (e.g. on a sunny day) produces visible cast shadows. Although the image becomes more cluttered by the addition of shadow-boundary edges (compare left and right close-up discs in figure 1*b*), this shading information could be beneficial as another source of depth information to promote image segmentation. In addition, the disruptive effects of edge enhancement might depend on the illumination conditions: the darker edge-abutting regions within edge-enhanced patterns resemble the cast shadows at object boundaries under directional illumination. Thus, under directional illumination, edge enhancement might provide more effective camouflage than under ambient illumination.

To the best of our knowledge, ours is the first study to investigate the interaction between illumination and disruptive coloration, including edge enhancement. However, previous work provides evidence that both cast shadows and shading across an object's surface are important determinants of crypsis. Both counter-shading and counter-illumination rely on the fact that illumination tends to come from above [1,29]. Counter-shaded animals including deer, goats, caterpillars and sharks are darker on their dorsal, illumination-facing surfaces and brighter on their undersides. This pattern of pigmentation counteracts the shading induced by overhead illumination and could improve the animal's background matching and/or make their three-dimensional shape harder to discern [2,3,30]. Behavioural evidence (using shaded ‘caterpillars’) suggests that counter-shading does improve crypsis in the natural environment [31]. In addition, many underwater animals including some sharks, fishes, squid and various crustaceans, use bioluminescence to emit downward illumination. This counter-illumination reduces their visual silhouette when viewed from below, and minimizes the shadow cast on the animals or surfaces below [32,33].

In the current study, human observers were asked to locate snake targets embedded in leafy backgrounds (figure 1*b*). We analysed response time (RT) as a function of edge enhancement, scene illumination and binocular depth information. We hypothesize that: (i) the presence of edge enhancement will disrupt target localization; (ii) this disruptive effect of edge enhancement may be reduced under stereoscopic viewing; (iii) search may facilitated by the cast shadows available under direct illumination; and (iv) edge enhancement may be more effective under direct illumination.

## 2. Methods

### (a) Participants

We determined that to detect an effect of edge enhancement similar in magnitude to that reported previously [13], with 90% power, we required eight participants. However, to detect an interaction between edge enhancement and disparity would require 26 observers (MorePower 6.0; [34]) under the hypothesis that the presence of disparity halves the effect of edge enhancement. Accordingly, 30 participants (17 females, aged 18–26) with normal or corrected-to-normal vision completed the experiment. Each participant gave informed prior consent and the Ethics Committee at the University of Southampton approved the study.

### (b) Stimuli

The colours for the snakes and leaves were sampled from forest scenes in the Southampton-York Natural Scenes (SYNS) dataset [1]. Four deciduous forest scenes (numbers 4, 19, 42, 45) and four coniferous forest scenes (15, 26, 47, 61) were used. Within each scene, 100 red, green, blue (RGB) values were sampled at random from locations below the horizon, resulting in 800 RGB triplets.

Stimuli were rendered in Blender 2.76 (Blender Foundation; blender.org). To create a unique leaf pattern for each trial, 4500 replicates of a three-dimensional leaf-shaped mesh were ‘dropped’ onto a brown background plane, using the rigid body physics simulation in Blender. The leaf mesh was formed by a Bezier curve and its coplanar reflection, before being curved in a direction orthogonal to the long (reflection) axis. Leaf colours were randomly sampled from the SYNS RGB triplets and surface shading was computed using interpolation between vertices. For each leaf, an initial position was randomly selected, bounded in *X* and *Y* by the background dimensions and in the range (5.0–7.0 cm) in *Z*. Each leaf was then rotated around the *Z*-axis through a randomly determined angle, before being dropped onto the ground plane, where leaves settled on top of each other up to a maximum height of approximately 1.5 cm. Subsequently, a target snake was dropped into the scene, from a height of 12 cm and a random *XY* position and *Z*-axis orientation. The physics model ensured that the snake rested on, rather than floated above the leaves; the snake was not always the closest object in the scene. The snake had an elliptical cross section that was constant (0.4° wide × 0.02° tall) other than the slightly wider ‘head’ and tapering ‘tail’ regions.

Snake textures were composed of two base colours that differed in both luminance and hue. First, the SYNS-based RGB colours were scaled to the range [0, 1] before conversion to hue, saturation, value colour space [35]. Colours with high or low luminance (greater than 0.75 or less than 0.25 on the value dimension) were excluded, to allow subsequent edge enhancement. To provide sufficient contrast in luminance and hue, two colours were randomly selected from the centile ranges 10–45 and 55–90, across all samples with a saturation value of more than 0.3. For each texture, random noise patterns with luminance in the range [0, 1] (figure 1*c*, (i)) were filtered with a Gaussian smoothing kernel (*σ* = 33). The resultant image was thresholded (i.e. values above or below 0.5 were rounded to 1 or 0), and then combined with the two base colours to create a texture without edge enhancement (figure 1*c*, iii). The edge enhancement pattern (figure 1*c*, iv) was defined by a linear luminance gradient in the range (±0.25, 0) applied to pixels lying to 0–10 pixels away from any edge. Edges were located using the Canny edge detector implemented in Matlab (MathWorks, Inc., Natick, MA). The enhancement pattern was added to the snake texture to create the edge-enhanced version (figure 1*c*, v).

Scenes were rendered under two different illumination conditions. In the ‘ambient’ condition, light was emitted uniformly from the upper hemisphere, whereas in the ‘directional’ condition, a collimated light source was positioned at 45° elevation and 350° azimuth, to produce distinct shadows.

Finally, we also manipulated the availability of naturalistic binocular disparities. Two virtual cameras, set 6.5 cm apart and located at a distance of 57 cm from the image plane captured separate views of the rendered scene. The resultant images were presented via a dual-monitor (two Asus PB328Q) single-bounce Wheatstone mirror stereoscope, with an effective viewing distance of 57 cm. This allowed the two eyes to view slightly different images on stereoscopic trials such that the pattern of binocular disparities provided information about the three-dimensional structure of the scene. On monoscopic trials, both eyes viewed the same image, and thus useful binocular information was eliminated. The full stimulus measured 39.64° by 28.74° visual angle. Each leaf element subtended 1.10° by 0.50° by 0.02°; the snake target was 4.00° by 0.40° by 0.02°.

### (c) Procedure

On each trial, the participant reported the location of the snake target (top left, top right, bottom left or bottom right), as quickly as possible, via key press. RTs and errors were recorded; trials terminated on response, or after 20 s. Experimental software was written in Matlab using Psychophysics Toolbox Version 3 [36,37]. Each observer completed 320 trials in random order, in a single session: 10 unique snake textures were presented in each of the spatial quadrants for all combinations of our three independent variables: (i) edge enhancement (present/absent), (ii) illumination (ambient/directional), and (iii) viewing condition (stereoscopic/monoscopic).

### (d) Analyses

One participant's data were removed because their error rate exceeded four standard deviations above the mean. Trials without a response (1.07% of trials), and those with an incorrect response (3.54%) were removed. After a reciprocal transformation to improve data normality, no outliers were detected (defined as RTs more than 3 s.d. from the mean). We collapsed each participant's data over the texture patterns and target quadrant, leaving three independent variables (edge enhancement, illumination, viewing condition) and a maximum of 40 data points in each condition. Reciprocal-transformed mean RTs were calculated for each condition, and submitted to a single 2 × 2 × 2 repeated measures ANOVA and simple effects *t*-tests in R [38]. ANOVA effect sizes were quantified using generalized eta squared ($\eta_{G}^{2}$), as recommended for repeated measured designs [39]. Data can be downloaded here: http://dx.doi.org/10.5258/SOTON/D0643.

## 3. Results

**Figure 3.**
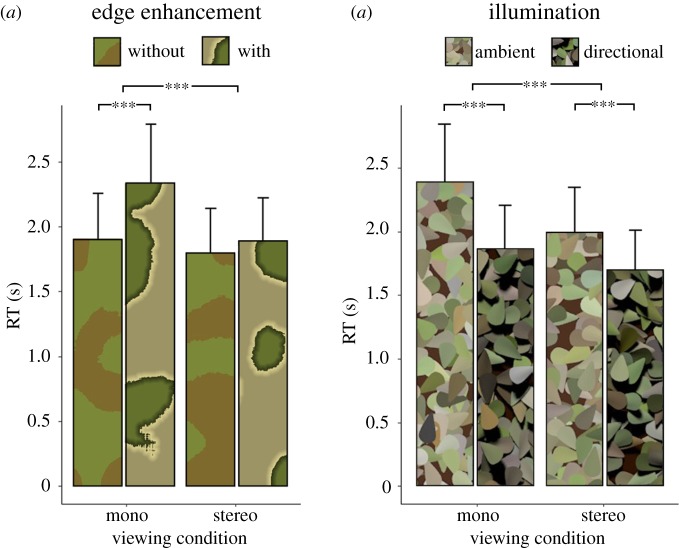
Results. (*a*) RTs for snake localization as a function of viewing condition and edge enhancement. (*b*) RTs as a function of illumination condition and viewing condition. Error bars give ±1 s.e. *** indicates *p* < 0.001 from ANOVA main effects and simple effects *t*-tests, as described in the text. (Online version in colour.)

Snakes with edge enhancement were significantly harder to find than those without, but only in the absence of valid binocular depth information, i.e. under monoscopic viewing. By contrast, under stereoscopic viewing, RTs were similar for targets with and without edge enhancement (figure 3*a*). These observations are supported by statistical analyses: edge enhancement increased RTs (main effect: *F*_1,28_ = 40.80, *p* < 0.001, $\eta_{G}^{2}=0.06$) and binocular depth information reduced reaction times (main effect: *F*_1,28_ = 59.94, *p* < 0.001, $\eta_{G}^{2}=0.07$) but these two factors strongly interacted: (*F*_1,28_ = 25.23, *p* < 0.001, $\eta_{G}^{2}=0.03$). Simple effects analyses confirmed that edge enhancement significantly increased RTs under monoscopic viewing (mean difference: 436.39 ms, *t*_28_ = −7.46, *p* < 0.001, *d* = 1.96), but not under stereoscopic viewing (mean difference: 96.40 ms, *t*_28_ = −2.03, *p* > 0.05, *d* = 0.53). In other words, edge enhancement was an effective camouflage strategy, but only in the absence of binocular depth information.

When scenes were rendered under directional illumination, such that both the leaves and snake produced strong cast shadows, snake targets were localized more quickly than under ambient illumination (mean difference: 416.74 ms, *F*_1,28_ = 38.08, *p* < 0.001, $\eta_{G}^{2}=0.14$). Illumination and binocular depth cues interacted such that the facilitatory effect of directional illumination (cast shadows present) was larger under monoscopic viewing than under stereoscopic viewing (*F*_1,28_ = 18.06, *p* < 0.001, $\eta_{G}^{2}=0.01,$ figure 3*b*). However, simple effects analyses confirmed that the facilitatory effect of directional lighting remained significant under both monoscopic (mean advantage: 540.75 ms, *t*_28_ = 6.37, *p* < 0.001, *d* = 1.67), and stereoscopic viewing (mean advantage: 309.97 ms, *t*_28_ = 5.11, *p* < 0.001, *d* = 1.34).

Edge enhancement was similarly effective under the two illumination conditions (no significant interaction between edge enhancement and illumination: *F*_1,28_ = 0.92, *p* > 0.05, $\eta_{G}^{2}=0.0003$). Finally, there was no significant three-way interaction between edge enhancement, illumination and viewing condition: *F*_1,28_ = 3.26, *p* > 0.05, $\eta_{G}^{2}=0.005$).

## 4. Discussion

Previous studies have reported a cryptic benefit of edge enhancement [4,13,40,41], similar to our results for disparity-absent conditions (left-most bars of figure 3*a*). Interestingly, within images of natural scenes, increased luminance and colour contrast at an edge reliably distinguish depth boundaries from other (non-depth) edges [42]. In other words, edge enhancement appears to work by mimicking the visual signals that, within the natural environment, signify the presence of object boundaries.

Under natural viewing of real scenes, binocular disparities provide reliable information about three-dimensional layout. When we gave observers this additional depth cue, edge enhancement was no longer effective. This can be understood via models of sensory cue integration [43,44]. The human visual system overcomes the noise and ambiguity of sensory signals (such as depth cues) by combining multiple cues. At shorter viewing distances, disparity information tends to dominate over pictorial cues such as texture and shading [45–48]. Thus, the reliable depth information provided by disparity over-ruled the depth boundaries implied by edge enhancement. Importantly, our work shows that depth information from binocular disparity can be processed alongside pictorial image cues to facilitate visual search, even in a complex, cluttered scene.

Previous work with traditional visual search paradigms suggests that binocular depth information can facilitate detection, but only when the depth of the target is known in advance: observers are able to selectively attend to, and search within, items belonging to the relevant depth plane [24,28]. In our scenes, target snakes were ‘dropped’ onto the leaves, and therefore tended to be stereoscopically closer to the observer than the average scene depth. Could our subjects have exploited binocular depth information by selectively searching within closer objects? Our data suggest otherwise: for targets without edge enhancement, there is very little advantage of stereoscopic viewing (figure 3*a*). Nakayama & Silverman [24] and Finlayson *et al*. [28] found a disparity advantage using arrays of non-overlapping objects organized into two distinct disparity-defined depth planes. By contrast, objects in our scenes were (more naturalistically) scattered in depth, and therefore did not elicit depth-defined perceptual grouping. Perceptual grouping according to depth may be required for more efficient, depth-limited search behaviour.

In the current study, stereoscopic viewing provided a substantial search benefit only in the edge enhancement condition. This suggests that a key benefit of binocular disparity lies in overcoming the disruptive effects of edge enhancement. In other words, while edge enhancement (falsely) suggests the presence of object boundaries, this misleading pictorial signal is ineffective when disparity information is available to reveal the true depth structure.

Recently, Sharman and co-workers [14] showed that, in the absence of stereoscopic depth information, edge enhancement not only disrupts target detection, but also target recognition. Furthermore, the disruptive effect on recognition persists when targets are poorly matched to their background, and thus quickly detected. An interesting question for further research is whether edge enhancement disrupts recognition when binocular depth cues are available. We suspect that, similarly to detection, stereopsis would also diminish the disruptive effects of edge enhancement on recognition.

Illumination also affected search: detection was faster under directional lighting, with cast shadows. Cast shadows provide depth information that may have facilitated depth segmentation, and the snake's elongated shadow may have provided a salient, low-level image cue to target location. The illumination effect was reduced in the presence of binocular disparity, suggesting that disparity and cast shadows provide partially redundant depth information. However, it is likely that the snake's long shadow also aided search; cast shadows did provide some additional benefit even when disparity-defined depth information was available. Edge enhancement was similarly effective in disrupting target detection in the two different illumination conditions. This is somewhat surprising: the ‘fake’ depth edges of the enhanced pattern are more similar to real depth boundaries under directional illumination, when cast shadows are present. For this reason, we hypothesized that edge enhancement might be more effective under directional illumination. However, our results suggest that the evolutionary advantage of edge enhancement is more general, and not restricted to particular illumination conditions.

Our findings suggest that binocular disparities are an important source of information for overcoming disruptive coloration, in line with early suggestions by Julesz [15] that stereopsis might counteract camouflage. Isbell [49], in her snake detection hypothesis, further argues that the evolution of orbital convergence and stereopsis in primates was driven by predation from venomous, camouflaged snakes. Motion parallax provides similar depth information to binocular disparity, but with significant drawbacks: the necessary head movements, such as vertical head-bobbing in gerbils [50] or side-to-side ‘peering’ head motion in insects such as crickets and locusts [51,52] take time and energy. Furthermore, the resultant retinal motion signals must be calibrated by distance moved, resulting in unreliable depth judgements, relative to stereopsis [53,54]. Most importantly, perhaps, these head movements will increase an animal's visibility. Stereopsis, by contrast, allows a predator or prey to recover reliable depth while remaining stationary and therefore less detectable [55].

It is now clear that stereopsis is not limited to primates, or even to mammals with front-facing eyes, but extends to many species including horses, sheep, cats, rabbits, owls, falcons, toads and praying mantises, and is likely to have evolved independently in mammals, amphibians, birds and insects [56,57]. One might speculate on the possible evolutionary pressures that drove the emergence of stereopsis in these different groups. Pettigrew [57] notes that within birds, stereopsis is seen among ‘perch and pounce’ predators (e.g. owls) that predate ground-dwelling prey. Stereopsis allows these birds to detect camouflaged prey without self-motion, and to accurately judge their own distance from the ground that they will hurtle towards. By contrast, solely aerial predators such as nightjars appear to lack stereopsis, but can rely on motion parallax for distance estimation, and are able to detect prey that are silhouetted against the sky.

Owls, toads and mantises have eyes that are fixed relative to the head. By contrast, humans and other primates use eye movements to scan a scene. This ocular motility complicates the recovery of absolute distance from stereopsis, because the disparity–distance relationship varies with eye position [58]. However, information about *relative* distance (including the presence of depth discontinuities at object boundaries) is preserved when eye position changes or is unknown. Thus, mobile-eyed predators have the advantage of being able to fixate different objects while remaining otherwise stationary, and preserving the relative disparity information required to segment camouflaged objects from their background. By contrast, fixed-eye stereopsis may have emerged in toads and praying mantises to support accurate striking (with tongue or forelegs) when food presents itself within striking range [56,59–61].

Other explanations for the evolution of stereopsis within primates have stressed benefits such as improved visuomotor coordination for arboreal primates leaping between branches [62,63] and more efficient foraging [64]. While our findings cannot discriminate between these competing theories, they are clearly consistent with the notion that stereopsis enhances the detection of camouflaged animals.

We have shown that the effectiveness of disruptive coloration, in the form of edge enhancement, depends critically on the availability of binocular depth information. What implications does this have for the evolution of camouflage patterns in the arms race between predators and prey? Edge enhancement should be more effective against viewers who lack stereopsis, and we might, therefore, expect edge enhancement to be more prevalent in species seeking to conceal themselves from such observers. For example, in a stereotypical predator–prey pairing, the prey species might benefit little from edge enhancement against a predator with substantial binocular overlap and good stereopsis. By contrast, the same predator might successfully employ edge enhancement to improve concealment against a prey species with little or no stereopsis. Some provisos, however: first, most animals have a variety of predator and prey species; the benefit of hiding from one or two lunch options may provide sufficient evolutionary pressure for edge enhancement to emerge, even if other prey or their own predators are stereoscopic. Second, the depth information provided by stereopsis becomes less reliable under certain conditions, such as when objects are partially occluded (e.g. by foliage) in a cluttered scene [65], or under weak illumination [66,67], or at large viewing distances [68]. Recent work suggests that high-contrast coloration can indeed have distance-dependent benefits: improving crypsis at large distances, but providing a warning (or sexual display) at close range [69,70]. Further work is needed to understand how variations in scene structure and viewing conditions modulate the relationship between binocular disparity and crypsis.

Finally, we ask whether there is any form of camouflage that could hinder stereopsis. One intriguing yet untested possibility is that the bright specular highlights on glossy surfaces might provide a cryptic advantage against stereoscopic observers [56]. Specular highlights are unlike textural patterns: the disparity-defined depth of a specular highlight differs from the depth of the reflecting surface, and varies with surface curvature [71,72]. In other words, specular highlights float in front of, or behind the glossy surface, and also move across the surface when it, or the observer moves. Thus, highlights could mislead observers about the position, shape or motion of glossy surfaces, and are indeed problematic for machine vision systems [73]. Conversely, rather than disguising an animal, recent evidence suggests that highlights on glossy, toxic beetles can serve as a heightened warning signal [74] but this question deserves further study.

## Data accessibility

Data are available at http://dx.doi.org/10.5258/SOTON/D0643.
